# Selective killing of human T-ALL cells: an integrated approach targeting redox homeostasis and the OMA1/OPA1 axis

**DOI:** 10.1038/s41419-018-0870-9

**Published:** 2018-08-01

**Authors:** Micol Silic-Benussi, Gloria Scattolin, Ilaria Cavallari, Sonia Minuzzo, Paola del Bianco, Samuela Francescato, Giuseppe Basso, Stefano Indraccolo, Donna M. D’Agostino, Vincenzo Ciminale

**Affiliations:** 1 Veneto Institute of Oncology IOV – IRCCS, Padua, Italy; 2 Department of Surgery, Oncology, and Gastroenterology, University of Padova, via Gattamelata 64, 35128 Padova, Italy; 3 Haemato-Oncology Division, Department of Woman and Child Health, University of Padova, via Giustiniani 3, 35128 Padova, Italy; 4 Department of Biomedical Sciences, University of Padova, via Ugo Bassi 58/B, 35131 Padova, Italy

## Abstract

Approximately 20% of pediatric T-cell acute lymphoblastic leukemia (T-ALL) patients are currently incurable due to primary or secondary resistance to glucocorticoid-based therapies. Here we employed an integrated approach to selectively kill T-ALL cells by increasing mitochondrial reactive oxygen species (ROS) using NS1619, a benzimidazolone that activates the K^+^ (BK) channel, and dehydroepiandrosterone (DHEA), which blunts ROS scavenging through inhibition of the pentose phosphate pathway. These compounds selectively killed T-ALL cell lines, patient-derived xenografts and primary cells from patients with refractory T-ALL, but did not kill normal human thymocytes. T-ALL cells treated with NS1619 and DHEA showed activation of the ROS-responsive transcription factor NRF2, indicating engagement of antioxidant pathways, as well as increased cleavage of OPA1, a mitochondrial protein that promotes mitochondrial fusion and regulates apoptosis. Consistent with these observations, transmission electron microscopy analysis indicated that NS1619 and DHEA increased mitochondrial fission. OPA1 cleavage and cell death were inhibited by ROS scavengers and by siRNA-mediated knockdown of the mitochondrial protease OMA1, indicating the engagement of a ROS-OMA1-OPA1 axis in T-ALL cells. Furthermore, NS1619 and DHEA sensitized T-ALL cells to TRAIL-induced apoptosis. In vivo, the combination of dexamethasone and NS1619 significantly reduced the growth of a glucocorticoid-resistant patient-derived T-ALL xenograft. Taken together, our findings provide proof-of-principle for an integrated ROS-based pharmacological approach to target refractory T-ALL.

## Introduction

Pediatric T-cell acute lymphoblastic leukemia (T-ALL) is an aggressive neoplasm of precursor T-cells^1^. Despite significant advances in treatment, approximately one out of five patients exhibit primary or secondary resistance to current therapies^2,3^, which include glucocorticoids as a key component; indeed, the overall clinical outcome depends on the initial response to glucocorticoids^4,5^.

Investigations of the genetics of T-ALL cells have identified a wide variety of mutations affecting several oncogenic pathways^6–8^. As more than 60% of T-ALL patients harbor activating mutations of *NOTCH1*^9,10^, therapeutic strategies aimed at blocking Notch signaling have been proposed.

In the present study, we used a novel approach to selectively sensitize T-ALL cells to apoptosis by increasing their levels of mitochondrial reactive oxygen species (mtROS). Intracellular ROS are tightly regulated second messengers that affect several signal transduction pathways controlling cell turnover^11^. Oncogenic pathways commonly activated in cancer cells drive a conspicuous increase in production of ROS^11,12^. Consequences of increased ROS include activation of the transcription factor NRF2 [nuclear factor (erythroid-derived 2)-like 2], which provides a powerful negative feedback loop by upregulating the expression of a wide array of antioxidant genes. Cancer cells frequently exhibit an imbalance between ROS accumulation and antioxidant defenses that results in a high setpoint of ROS^13^, which is close to the threshold beyond which macromolecular damage is produced and cell death pathways are engaged. Therefore, tumor cells are predicted to be more vulnerable than their normal counterparts to treatments that impinge on ROS homeostasis^11^ and particularly to agents that blunt antioxidant systems. Among these, the pentose phosphate pathway (PPP) has a central role as a source of NADPH, the universal electron donor used to replenish the reducing capacity of ROS scavengers.

Our approach builds on our previous studies of p13, a mitochondrial protein coded by human T-cell leukemia virus type 1 (HTLV-1), the causative agent of adult T-cell leukemia^14,15^. We showed that p13 increases mitochondrial K^+^ permeability and ROS production and triggers death in transformed T-cell lines but not in normal T-cells^16,17^. To mimic p13 activity, we used NS1619, an opener of the mitochondrial large conductance K^+^ (BK) channel^18,19^. To interfere with ROS scavenging pathways, we employed dehydroepiandrosterone (DHEA), which inhibits glucose-6-phosphate dehydrogenase, the rate-limiting enzyme of the PPP^20^. Pilot experiments were performed on TALL-1^21^, a cell line that was stabilized from a patient with refractory T-ALL. Key findings were validated using (i) additional T-ALL cell lines CEM, Jurkat, and Molt-3, (ii) patient-derived xenografts explanted from NOD/SCID mice (PDX; Table S1)^22^, and (iii) primary samples from T-ALL patients (Table S2). Normal human thymocytes were used as controls. Results showed that NS1619 and DHEA induced mtROS, feedback activation of NRF2, and triggered death of T-ALL cells. The treated cells showed changes in mitochondrial morphology indicative of increased fission as well as increased cleavage of OPA1 (optic atrophy 1), a protein that regulates mitochondrial dynamics and apoptosis^23^. OPA1 cleavage was dependent on both ROS and the metallopeptidase OMA1^24,25^. NS1619 and DHEA also sensitized T-ALL cells to death induced by TRAIL (tumor necrosis factor-related apoptosis-inducing ligand)^26^. In contrast, normal human thymocytes were resistant to killing by NS1619, DHEA, and TRAIL.

## Results

### The potassium channel opener NS1619 and the PPP inhibitor DHEA increase mtROS and activate NRF2

**Fig. 1:**
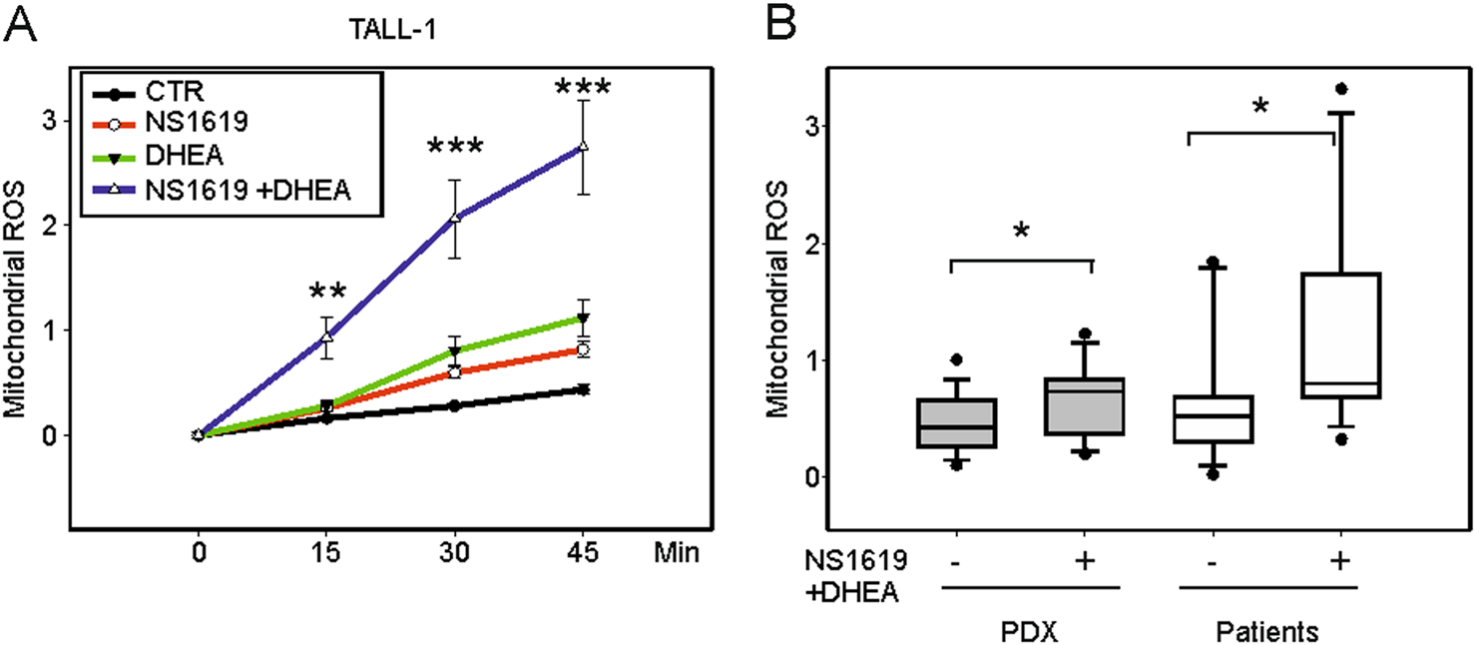
NS1619 and DHEA increase mtROS. mtROS accumulation was measured using MitoSOXRed and flow cytometry and expressed as ΔF/F_0_ ratios (see Materials and Methods). **A** mtROS accumulation in TALL-1 cells (three independent experiments, three replicates each) without treatment (CTR, black line) and in the presence of NS1619 (red), DHEA (green), or NS1619 + DHEA (blue). **B** mtROS accumulation in PDX after 45 min (gray boxes, left, *N* = 5, 3 replicates each) and primary cells from T-ALL patients (white boxes, right, *N* = 7, 2 replicates each) in the absence ( − ) or presence ( + ) of NS1619 + DHEA. Mean values and SE bars are shown

With the aim of selectively killing T-ALL cells, we used an integrated approach to increase mitochondrial ROS and depower ROS-scavenging pathways. To this end, we employed NS1619, a benzimidazolone derivative that opens the large conductance Ca^2+^-activated K^+^ (BK) channel, which increases electron transport chain (ETC) activity, resulting in increased mtROS generation^18,19^. As shown in Fig. 1A, NS1619 increased the rate of mtROS accumulation over a 45 min time course in the TALL-1 cell line (red line). Inhibition of the PPP with DHEA also increased the rate of mtROS accumulation (green line) and significantly enhanced the effect of NS1619 (blue line).

NS1619 + DHEA also significantly increased the rate of mtROS accumulation in PDX (*N* = 5) and primary samples from T-ALL patients (*N* = 7) (Fig. 1B). These effects were also observed in other T-ALL cell lines (Molt-3, CEM, and Jurkat) and, to a lesser extent, in normal human thymocytes (Fig. S1A). Isobologram analysis using the Chou and Talalay equation^27^ (see Supplemental Materials and Methods) revealed that the effects of the association of NS1619 and DHEA on mtROS production were highly synergistic (Fig. S1B).

Analysis of ROS at 24 and 48 h after treatment revealed that although cells treated with DHEA or DHEA + NS1619 continued to accumulate ROS, NS1619 alone did not have long-term effects (Fig. S1C).

**Fig. 2:**
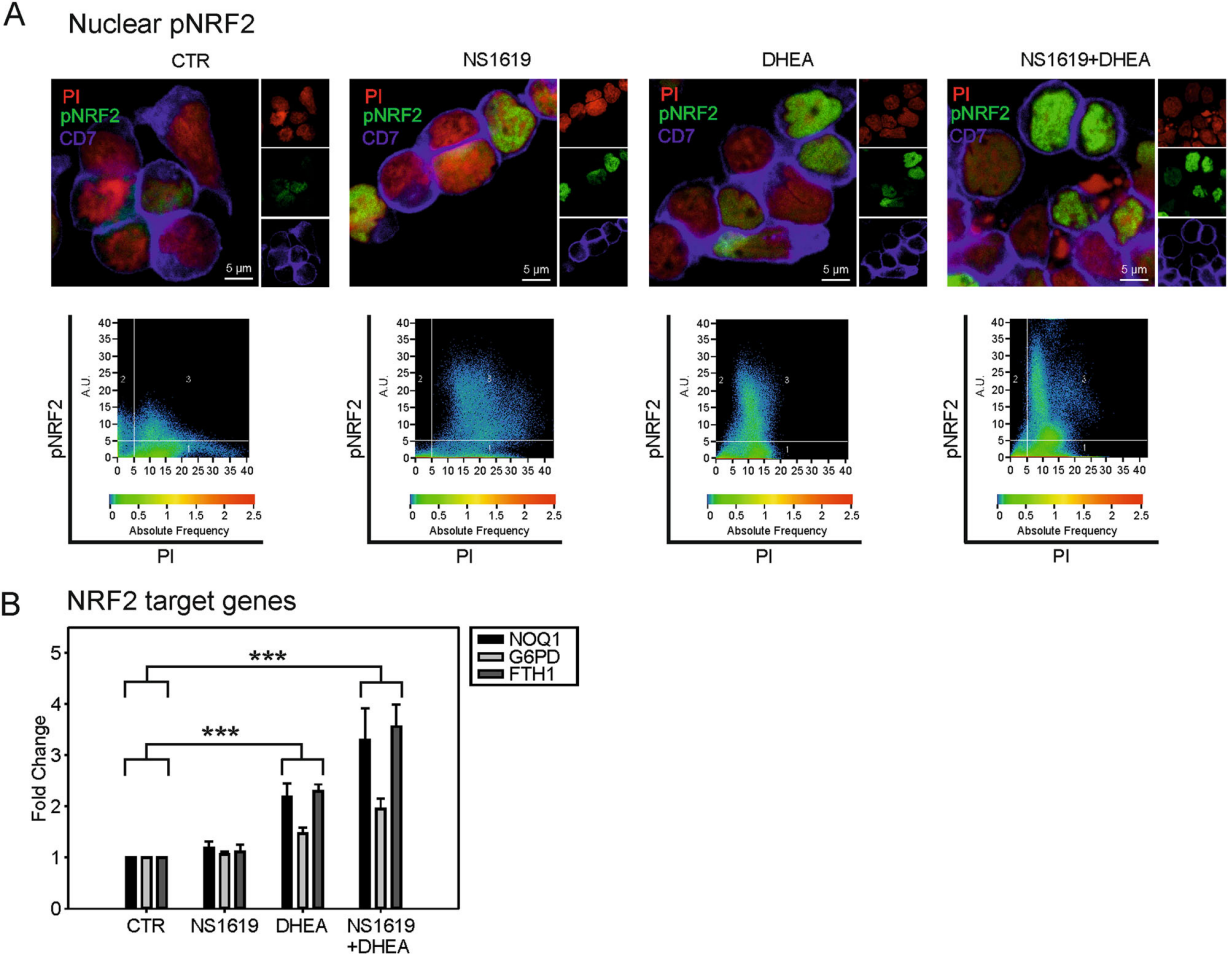
NS1619 and DHEA activate NRF2. **A** Immunofluorescence of TALL-1 cells following 24 h of treatment with NS1619 and DHEA. An anti-phosphoS40-NRF2 antibody was used to detect pNRF2 (green). Propidium iodide (PI, red) and APC-conjugated anti-CD7 antibody (blue) were used as a nuclear and membrane markers, respectively. Lower panels show the pixel dot plots of the pNRF2 and PI signals in representative fields. **B** qRT-PCR of NRF2 target genes NQO1 (NAD(P) H:quinone oxidoreductase 1), G6PD (glucose-6-phosphate dehydrogenase), and FHT1 (ferritin heavy chain 1) after 24 h of treatment of TALL-1 cells with NS1619 and DHEA. β2-microglobulin (B2M) was used as a housekeeping mRNA and all values were scaled against untreated cells (CTR). Mean values and SE bars from seven experiments are shown

Increased ROS levels are known to activate NRF2, the master regulator of antioxidant pathways, through oxidation/inactivation of its negative regulator KEAP1 (Kelch-like ECH-associated protein 1)^28,29^ and through phosphorylation of NRF2 on serine 40 by atypical PKCs, leading to its nuclear translocation^12,29^. The status of NRF2 activation in TALL-1 cells was investigated by fluorescence microscopy on cells labeled with an anti-phospho-NRF2 antibody (pNRF2); results indicated that NS1619 and DHEA significantly increased the levels of nuclear pNRF2 (Fig. 2A, Fig. S2). Moreover, results of quantitative reverse transcription-PCR (qRT-PCR) showed that DHEA alone or in combination with NS1619 significantly increased the expression of NRF2 target genes (Fig. 2B).

### NS1619 and DHEA selectively kill T-ALL cells

We next tested the effects of NS1619 and DHEA on cell death. Pilot experiments in TALL-1 cells showed that NS1619 and DHEA increased the percentage of Annexin V/propidium iodide (PI)-positive cells, a staining pattern indicative of late apoptosis (Fig. S3). In subsequent experiments, cell death was evaluated by PI staining and flow cytometry 24, 48, and 72 h after treatment, and specific cell death values were calculated (see Materials and Methods) to allow comparison of the results of different experiments.

**Fig. 3:**
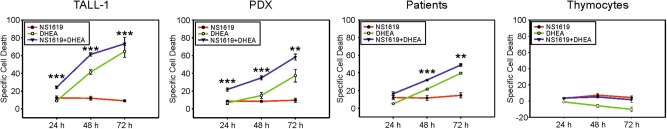
NS1619 and DHEA kill T-ALL cells. Specific cell death (see Materials and Methods) of TALL-1 (three experiments, three replicates each), PDX (three samples, three replicates each), primary cells from T-ALL patients (three patients, three replicates each), and normal human thymocytes (four donors, three replicates each) after 24, 48, and 72 h of treatment with NS1619 (red), DHEA (green), or NS1619 + DHEA (blue); mean values and SE bars are shown; lower panels show *p*-values for the indicated pairwise comparisons

Results showed that NS1619 alone (Fig. 3, red lines) exerted a modest pro-death effect in the first 24 h that was blunted at later time points, most likely due to activation of NRF2. Interestingly, these results closely mirrored the long-term effects on mtROS (Fig. S1C). DHEA (Fig. 3, green lines) and DHEA + NS1619 (Fig. 3, blue lines) induced a substantial increase in death in all the T-ALL cell lines, in PDX explants (*N* = 3), and in primary T-ALL samples (*N* = 3), but did not kill normal primary thymocytes (*N* = 4;) and induced only a modest increase in death of peripheral blood mononuclear cells (PBMCs) (Fig S4).

Isobologram analysis indicated that NS1619 and DHEA had an additive effect on death of TALL-1 cells (data not shown). The observation that DHEA alone induced cell death despite activating NRF2 (Fig. 2) is consistent with its ability to block NADPH production by the PPP, thus crippling the reducing capacity of scavenger pathways.

The importance of blocking antioxidant responses to achieve high levels of cell death was supported by the finding that NS1619-induced killing of TALL-1 cells was enhanced by glucose deprivation in the presence of pyruvate, which curtails PPP fueling while maintaining ETC activity (Fig. S5). This effect was counteracted by the ROS scavenger *N*-acetyl-l-cysteine (NAC, Fig. S5), demonstrating its ROS dependence.

### Cell death induced by NS1619 and DHEA is mediated by engagement of the OMA1-OPA1 axis

The observation that NS1619 + DHEA increased mtROS prompted us to search for mitochondrial ROS-sensitive target(s) involved in the cell death induced by these drugs. We focused on OPA1, a dynamin-related GTPase of the inner mitochondrial membrane that has a key role in mitochondrial dynamics and apoptosis^23,30,31^. The function of OPA1 is controlled through processing by mitochondrial proteases including OMA1, whose activity is known to be influenced by ROS^25,32,33^. We therefore tested whether NS1619 and DHEA affect the cleavage of OPA1.

**Fig. 4:**
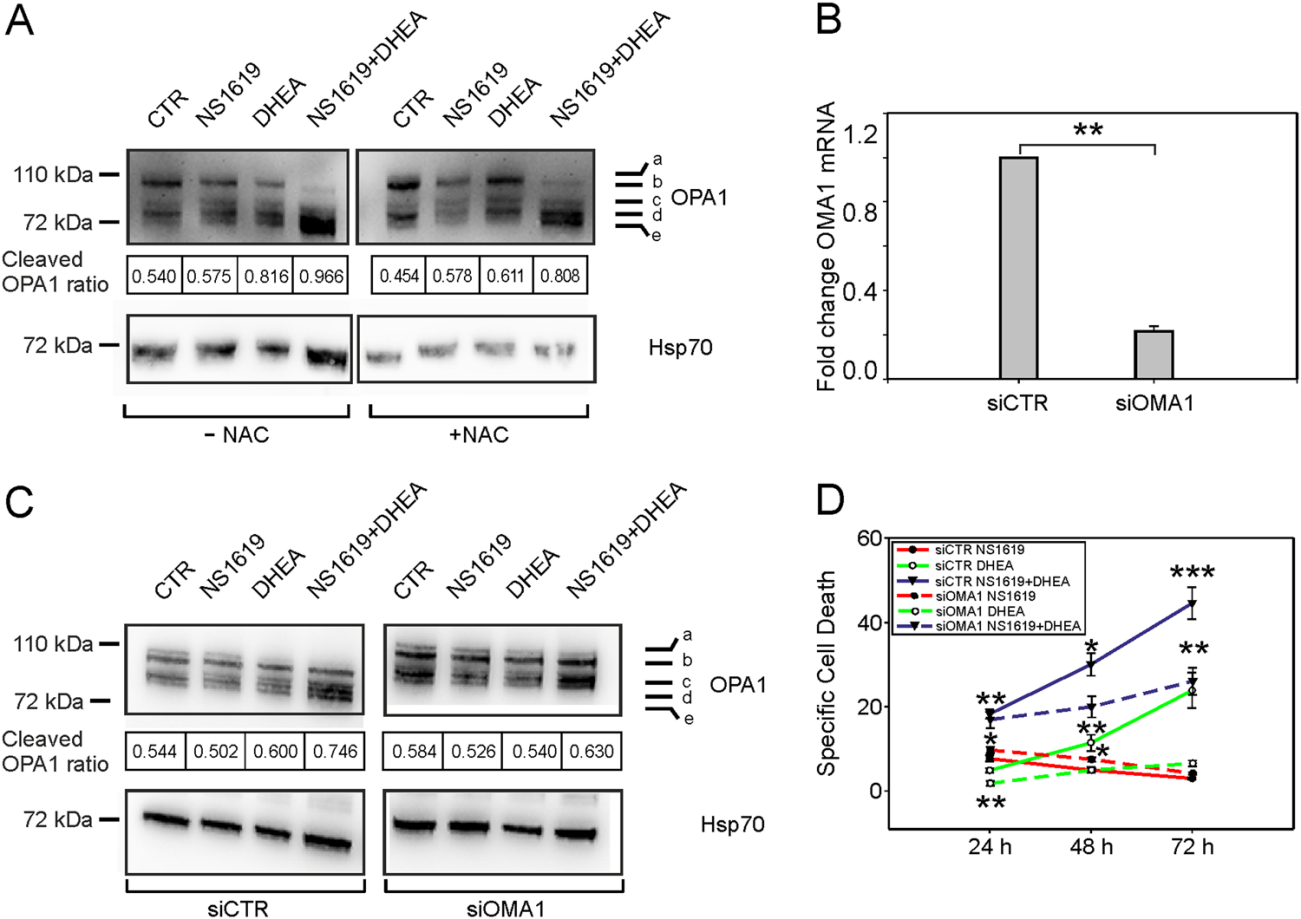
Effects of NS1619 and DHEA on OPA1. **A** Immunoblot of a representative experiment showing the five major OPA1 isoforms (**A**–**E**) in TALL-1 cells after 24 h of the indicated treatments. *Cleaved OPA1 ratios* (see Materials and Methods) are shown below the blots. NAC *N*-acetyl-l-cysteine. **B** Quantitative RT-PCR analysis of OMA1 mRNA expression in TALL-1 cells after electroporation with siRNA specific for OMA1 (siOMA1) or a control siRNA(siCTR). β2-microglobulin (B2M) was used as a housekeeping mRNA and values were scaled against the CTR. Mean values and SE bars from three experiments are shown. **C** Immunoblots of a representative experiment showing the five major OPA1 isoforms in TALL-1 cells transfected with control siRNA (left) or OMA1 siRNA (right) after 24 h of the indicated treatments. *Cleaved OPA1 ratios* (see Materials and Methods) are shown below the blots. **D** Specific cell death of TALL-1 cells after electroporation with control siRNA (continuous lines) or OMA1-specific siRNA (dashed lines) followed by treatment with NS1619 (red), DHEA (green) or NS1619 + DHEA (blue). Mean values of specific cell death and SE bars from three independent experiments are shown

Figure 4A shows a representative immunoblot to detect the 5 main isoforms of OPA1 in TALL-1 cells, with bands labeled a and b representing full-length OPA1, and bands c, d, and e corresponding to processed forms. The amount of OPA1 cleavage was measured as the *cleaved OPA1 ratio* (see Materials and Methods). After 24 h of treatment, NS1619 and DHEA alone or in combination induced a relative increase in the cleaved OPA1 ratio. This effect was confirmed in the other T-ALL cell lines (Fig S6A-C) and in PDX (Fig. S6D). NS1619 + DHEA also reduced the overall expression of OPA1 mRNA measured by qRT-PCR (Fig. S6E), suggesting a ROS-mediated control of OPA1 expression.

The effects of NS1619 and DHEA on OPA1 cleavage were less evident in the presence of NAC (Fig. 4A), indicating their ROS dependence and suggesting the involvement of OMA1^24,25^. To test this hypothesis, we analyzed the effects of NS1619 and DHEA in TALL-1 cells following small interfering RNA (siRNA)-mediated knockdown of OMA1, which resulted in an 80% reduction of its mRNA (Fig. 4B). Interestingly, both OPA1 cleavage (Fig. 4C) and cell death (Fig. 4D) induced by NS1619 and DHEA were reduced in OMA1-silenced cells. Consistent with these findings, the cleavage of OPA1 and induction of apoptosis (measured as cleaved Caspase 3) in response to NS1619 + DHEA was abrogated in fibroblasts obtained from OMA1^−/−^ mice^24,33^ (Fig. S7).

**Fig. 5:**
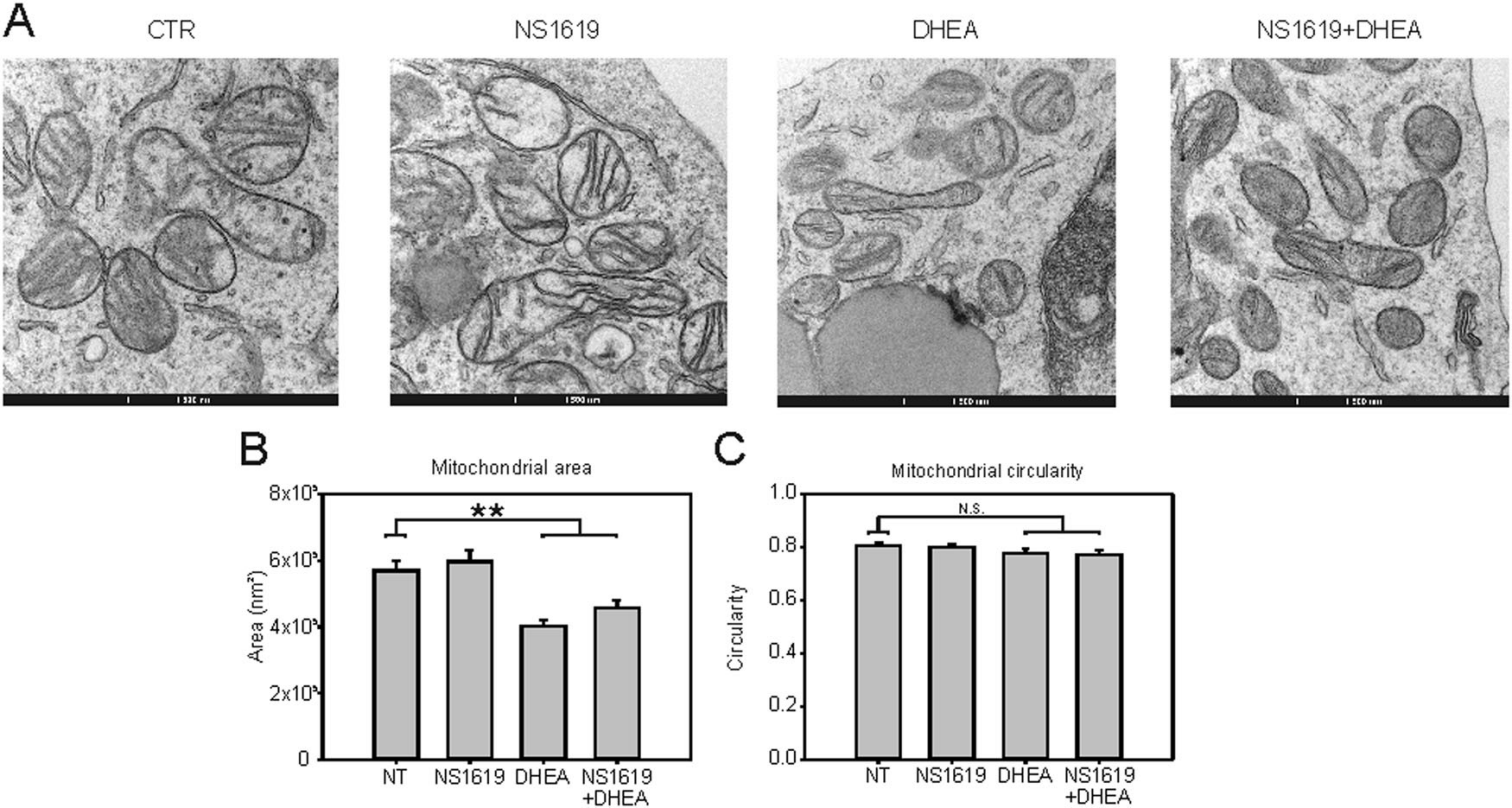
Effects of NS1619 + DHEA on mitochondrial morphology. **A** Representative images of electron microscopy analysis showing mitochondria of TALL-1 cells after 24 h of treatment with NS1619 and DHEA. **B**, **C** Quantification of mitochondrial area (**B**) and circularity (**C**) (see Materials and Methods) in TALL-1 cells subjected to the indicated treatments for 24 h. The graph shows mean values and SE bars from analysis of at least 130 mitochondria per treatment

OPA1 controls mitochondrial function and dynamics in part by promoting mitochondrial fusion^23–25,31^. We therefore tested whether the increased OPA1 cleavage induced by NS1619 and DHEA was accompanied by a change in mitochondrial morphology. Results of transmission electron microscopy analysis (Fig. 5) showed that 24 h of treatment of TALL-1 cells with DHEA alone or in combination with NS1619 significantly reduced the mean mitochondrial area, whereas circularity was unchanged, indicating a relative increase in mitochondrial fission, a finding that is consistent with a decrease in OPA1 function following its processing by OMA1.

### NS1619 and DHEA sensitize T-ALL cells to TRAIL-induced death

We next investigated whether NS1619 and DHEA sensitize T-ALL cells to killing by TRAIL, which induces apoptosis through tBid-mediated opening of the Bax/Bak pore^26,34–37^.

**Fig. 6:**
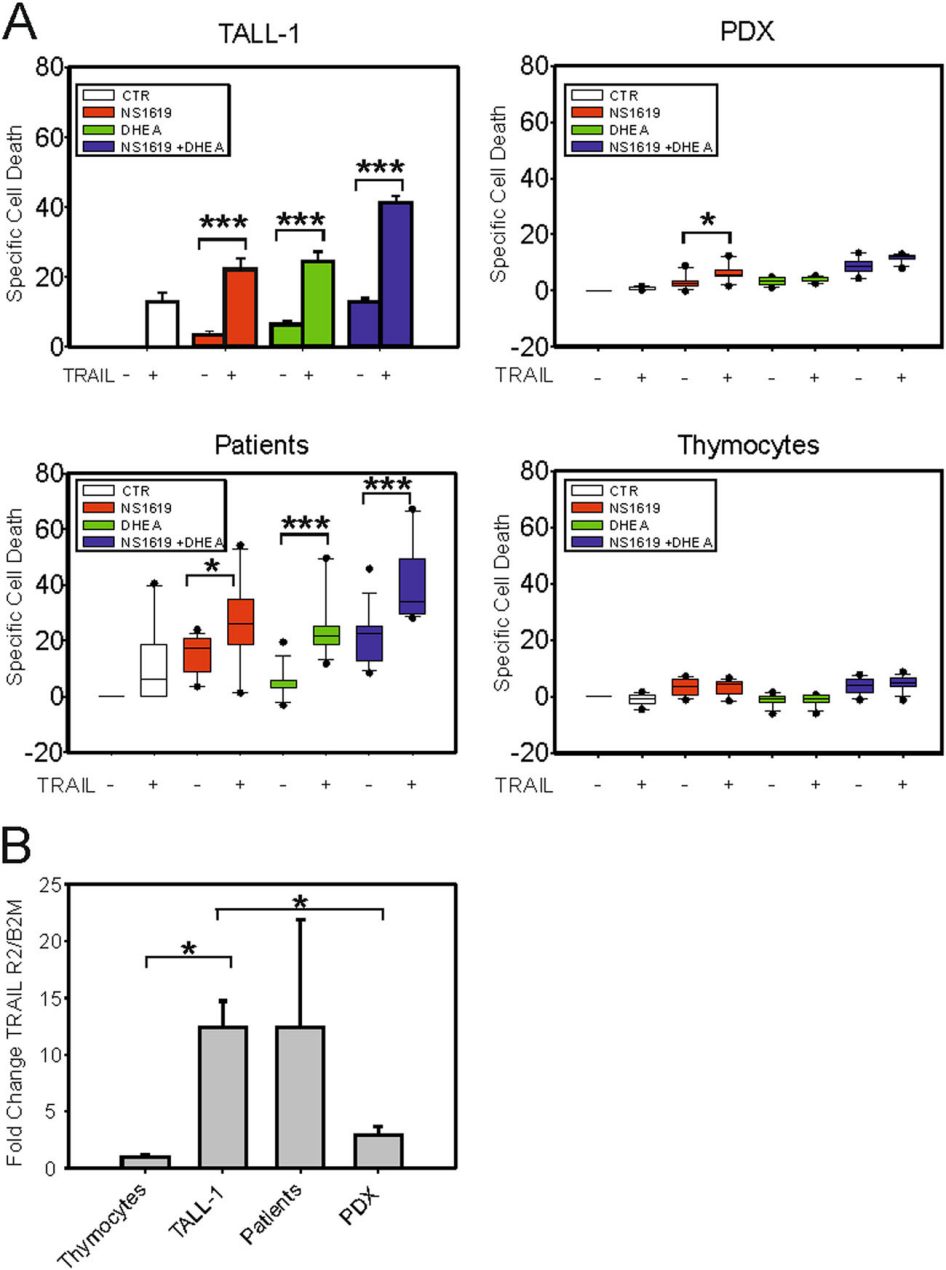
NS1619 and DHEA sensitize T-ALL cells to TRAIL. A Specific cell death upon 24 h of treatment with NS1619, DHEA or NS1619 + DHEA in the absence ( − ) or presence ( + ) of TRAIL in TALL-1 cells (three independent experiments, three replicates each), PDX (*N* = 4, three replicates each), primary T-ALL samples (*N* = 7, three replicates each), and primary thymocytes (*N* = 4, three replicates each). Mean values and SE bars are shown. **B** Quantitative RT-PCR analysis of TRAIL-R2 mRNA expression in normal thymocytes (*N* = 3), TALL-1 cells (8 repeats), primary T-ALL samples (*N* = 7), and PDX (*N* = 5). β2-microglobulin (B2M) was used as a housekeeping mRNA and values were scaled against those obtained for thymocytes. Mean values and SE bars are shown

As shown in Fig. 6A, TALL-1 cells exhibited a modest response to 24 h of treatment with TRAIL alone, but showed more substantial death when TRAIL was combined with NS1619 + DHEA. Similar results were obtained in Molt-3 and Jurkat cells, whereas CEM cells were refractory to TRAIL (Fig. S8A-C, upper panels). qRT-PCR analysis showed that NS1619 + DHEA induced a significant upregulation of TRAIL-receptor-2 (R2) mRNA in TALL-1 cells (Fig. S9A). Interestingly, TRAIL-R2 mRNA levels were very low in CEM cells (Fig. S9B), which might explain their resistance to TRAIL. Consistent with cell death results, treatment of TALL-1 cells with NS1619, DHEA, and TRAIL induced mitochondrial depolarization, release of cytochrome *c* from mitochondria, and cleavage of Caspase 3 (Fig. S10), which are key events in the intrinsic apoptotic pathway. These effects were accompanied by increased cleavage of PARP1 (poly ADP-ribose polymerase 1), a substrate of effector caspases (Fig. S8A-C, lower panels).

Experiments on PDX explants (*N* = 4, Table S1) showed that NS1619 + DHEA efficiently induced PARP1 cleavage (Fig. S11) and a significant increase in death (Fig. 6A). However, the response of the PDX to NS1619 + DHEA was not substantially affected by TRAIL (Fig. 6A).

In contrast to PDX, primary T-ALL cells from refractory patients (*N* = 7) were highly sensitive to TRAIL, with almost 40% specific cell death after 24 h; interestingly, this effect was further enhanced by NS1619 and DHEA, with > 60% specific cell death after 24 h (Fig. 6A). Notably, neither NS1619, DHEA, TRAIL, nor combinations of these drugs induced significant cell death in normal thymocytes (Fig. 6A).

The apparent insensitivity of the PDX and normal thymocytes to TRAIL was consistent with their low level of expression of TRAIL-R2 compared with primary samples from T-ALL patients, which exhibited much higher, although quite variable, TRAIL-R2 expression (Fig. 6B).

We reasoned that the observed sensitivity of T-ALL cells to NS1619 and DHEA might be due to different levels of expression of OMA1 or OPA1. Results of qRT-PCR analyses revealed variations in the expression levels of these transcripts among T-ALL cell lines, PDX and primary T-ALL samples, which, however, did not correlate with their response to NS1619 and DHEA. We also did not observe a significant difference in the expression levels of these genes between normal thymocytes and primary T-ALL or PDX samples (Fig. S12A, B). In addition, gene expression profile data available on the Leukemia Gene Atlas platform did not show significant differences in OMA1 or OPA1 expression between T-ALL primary samples (*N* = 174) and non-leukemic bone marrow samples (*N* = 73) (Fig. S12C, D).

### Treatment with NS1619 plus dexamethasone reduces PDX growth in vivo

We next investigated the effectiveness of NS1619 and DHEA in NOD/SCID mice inoculated with PDX19, which was derived from a patient with refractory T-ALL. Unfortunately, preliminary experiments showed that DHEA was toxic for the mice; in addition, the PDX, including PDX19, were insensitive to TRAIL (see Fig. 6). The in vivo experiments were therefore restricted to testing the ability of NS1619 to enhance the effects of dexamethasone (DEX)^2,4^. Mice were inoculated in the tail vein with PDX19 cells engineered to express luciferase and then treated with drugs as described in the Supplemental Methods. Tumor growth was monitored once a week by measuring the percentage of CD5 + /CD7 + tumor cells in the peripheral blood and by in vivo imaging to detect luciferase-expressing cells; for ethical reasons, mice were sacrificed when circulating CD5 + /CD7 + cells reached 40% of the total PBMC population.

**Fig. 7:**
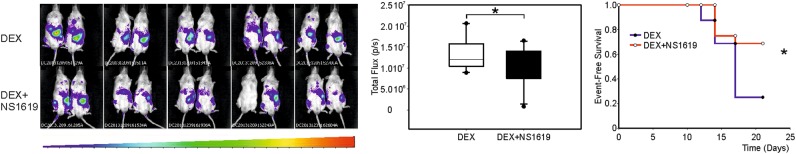
In vivo effects of NS1619 and dexamethasone. Xenogen images of mice to detect luciferase-engineered PDX19 cells; shown are images obtained at 21 days (left). Total photon/second flux (mean values and standard error) measured by Xenogen imaging in the two experimental groups at 21 days (middle). Kaplan–Meyer plot of event-free survival (right) in which an event was defined when PDX19 cells (i.e., CD5 + CD7 + ) detected by flow cytometry were at least 40% of the total PBMC

As single agents, NS1619 and DEX did not significantly affect the growth of PDX19 in the mice (data not shown). We thus set up an experiment on two groups of 10 mice each to test whether the combination of NS1619 and DEX would yield a better result compared to the treatment with DEX alone. Results of in vivo imaging analyses indicated that the dimensions of the tumor masses were smaller in mice treated with DEX + NS1619 compared with mice treated with DEX alone (Fig. 7, left and middle panels). Kaplan–Meyer analysis (Fig. 7, right panel) demonstrated that the event-free survival (where an event was defined when leukemic cells were 40% of the PBMC) of the DEX + NS1619-treated group was significantly increased compared with the group that received DEX alone.

## Discussion

The present study was aimed at priming refractory T-ALL cells to apoptosis by altering their mitochondrial redox homeostasis. Treatment of T-ALL cells with NS1619, an opener of the mitochondrial large conductance Ca^2+^-activated K^+^ (BK) channel that is known to produce an inward K^+^ current in polarized mitochondria and an enhancement of respiratory chain activity^38^, resulted in a significant increase in mtROS accumulation (Fig. 1). The effects of NS1619 on mtROS were enhanced by PPP inhibition (either with DHEA, Fig. 1, or glucose deprivation, Fig. S5), which interferes with the synthesis of NADPH, the central electron source used by cells to replenish the reducing capacity of ROS-scavenging molecules. The importance of depowering scavenging pathways to increase the efficacy of ROS-producing treatments is supported by the observation that NS1619 and DHEA activated the negative feedback loop mediated by NRF2 (Fig. 2)^29^.

Isobologram analysis using the Chou and Talalay equation revealed that the effects of NS1619 and DHEA on ROS accumulation were highly synergistic (Fig. S1B), a finding that is consistent with the fact that the two drugs target two distinct pathways controlling ROS production and scavenging, respectively. On the other hand, the observation that NS1619 and DHEA exhibited an additive (non-synergistic) effect on cell death suggested that the ROS-enhancing effect of NS1619 and DHEA converge on the same death-promoting pathway.

NS1619 and DHEA induced death of T-ALL cell lines, PDX, and primary cells from T-ALL patients, but did not kill normal primary thymocytes (Fig. 3) and induced only a modest increase in death of PBMC (Fig. S4D), suggesting that this drug combination is not likely to induce major side effects if translated to the clinic. The NS1619-DHEA combination also substantially increased death induced by TRAIL, an effect that was highly specific for T-ALL cells (Fig. 6).

The finding that NS1619 induced ROS in the mitochondrial microdomain suggested the involvement of mtROS-sensitive targets. Our results show that increasing mtROS levels in T-ALL cells with NS1619 and DHEA induced cleavage of OPA1 (Fig. 4) and changes in mitochondrial morphology indicative of increased fission (Fig. 5). Results of knockout and knockdown experiments indicated that OMA1 had a major role in NS1619 + DHEA-induced cleavage of OPA1 (Fig. 4). It is noteworthy that the function of OPA1 is also controlled by the YME1L protease^32,33^, which however does not induce mitochondrial fragmentation or apotosis^39^. Our data also indicate that the increase in ROS (measurable after minutes) precedes the effects on mitochondrial depolarization and cell death (measurable after days).

The differential effect of NS1619 and DHEA in T-ALL cells vs. normal thymocytes could be explained in part by the status of the “ROS rheostat.” In fact, all T-ALL cell lines tested exhibited a considerably higher rate of mtROS accumulation in response to NS1619 and DHEA compared with normal thymocytes (Fig. 1). However, PDX and primary samples from T-ALL patients exhibited a relatively low rate of mtROS accumulation, arguing against a simple direct correlation between the rate of mtROS accumulation and the effects on cell death.

The response of T-ALL cells and normal thymocytes to NS1619 and DHEA may also be influenced by differential expression/activity of IF1 (ATPase inhibitory factor 1), a mitochondrial protein that, by inhibiting the reverse activity of the ATP synthase, maintains ATP levels favoring cell survival under oxidative stress and inhibits ROS-induced OPA1 cleavage through inhibition of OMA1 activity^40^.

The fact that DHEA is a steroid, together with our observations that DHEA is able to (i) rapidly increase mitochondrial ROS and (ii) influence mitochondrial shape, suggest that it could interact with TSPO (translocator protein), an outer mitochondrial membrane protein responsible for entry of cholesterol into mitochondria. TSPO, which is upregulated in many types of cancer, was also shown to regulate Ca^2+^ homeostasis and ROS production^41^, and to inhibit mitophagy through a VDAC1-mediated mechanism^42^. Further studies are needed to investigate the possible role of IF1 and TSPO in the effects of NS1619 and DHEA on T-ALL cells.

Although we observed that DHEA was toxic in NOD/SCID mice, this compound is currently used in humans^43,44^, suggesting that its translation to clinical use in T-ALL might be feasible. The toxicity of DHEA in NOD/SCID mice and the resistance of PDX to TRAIL precluded their testing in our preclinical setting. However, the modest, but significant effect of NS1619 when combined with DEX in vivo is encouraging and supports the proposal that ROS-dependent engagement of the OMA1-OPA1 axis combined with TRAIL may prove to be an effective strategy for treating refractory T-ALL, which poses a major clinical challenge at present.

## Materials and methods

### Cells and patients

All procedures involving patients, animals, and their care were authorized by the ethics committees of the University of Padova and the Italian Ministry of Health (Authorization number 894/2016-PR) in conformity with national and international laws (EEC Council Directive 86/609, OJ L 358, 12/12/1987). Primary T-ALL samples were obtained as bone marrow aspirates. Control thymocytes were isolated from thymus tissue of four children who underwent cardiac surgery. PBMCs were obtained from four healthy donors. Xenografts stabilized from primary T-ALL cells (PDX) were propagated in NOD/SCID mice as previously described^22^. The T-cell receptor and short tandem repeat profiles of the PDX matched those of the original T-ALL cells from the patients.

Cell lines were cultured in RPMI 1640 medium (Sigma-Aldrich) supplemented with 2 mM l-glutamine, 100 units/mL penicillin, 20 units/mL streptomycin (complete RPMI), and 10% fetal calf serum (FCS). Human thymocytes, PBMC, patients’ samples, and PDX were cultured in complete RPMI supplemented with 20% FCS.

### Drug treatments

Cells were treated with 25 μM NS1619 (Sigma-Aldrich), 100 μM DHEA (Sigma-Aldrich), and 50 ng/ml TRAIL (Alexis), as described in the figure legends. Where indicated, the cells were incubated with 500 μM NAC (Sigma-Aldrich) for 16 h before other drug treatments.

### ROS measurements

Mitochondrial ROS were measured by flow cytometry in the gate of living cells using MitoSOXRed (Life Technologies)^45,46^ (see Supplementary Materials and Methods). Changes in ROS were calculated as the difference between the mean fluorescence intensity (MFI) at each time point (*F*_X_) and the MFI of the initial time point (*F*_0_) divided by *F*_0_, which yielded the Δ*F*/*F*_0_ ratio [(*F*_X_ − *F*_0_)/*F*_0_].

### Immunofluorescence

Cells were labeled with antibodies against Phospho-Nrf2 and CD7 together with PI, and analyzed by confocal microscopy as described in the Supplemental Materials and Methods.

### Specific cell death

Cell death was evaluated by staining cells with PI followed by flow cytometry. Specific cell death was calculated using the formula [(% dead cells in the treated sample − % dead cells in the control sample)/(100 − % dead cells in control)] × 100.

### Immunoblotting

Protein lysates were separated by SDS/polyacrylamide gel electrophoresis, transferred to nitrocellulose, and probed with antibodies against OPA1, Caspase 3, PARP1, β-actin, Hsp70, and vinculin, followed by horseradish peroxidase-conjugated secondary antibodies as described in the Supplemental Materials and Methods. The *cleaved OPA1* ratio was calculated by dividing the signals of bands c, d, and e (see Fig. 4A) by the signals of all bands (see also Supplemental Materials and Methods).

### Electron microscopy

TALL-1 cells were treated for 24 h with compounds indicated in the text and then processed for electron microscopy to assess mitochondrial morphology as described in the Supplemental Materials and Methods.

### Quantitative RT-PCR

Total RNA was isolated, reverse-transcribed, and PCR-amplified with primer pairs to detect mRNAs coding for β2-microglobulin, TRAIL-R1, TRAIL-R2, OMA1, OPA1, and NRF2-responsive genes as described in the Supplemental Materials and Methods.

### RNA silencing experiments

Five million TALL-1 cells were electroporated with a siRNA specific for OMA1 or a control siRNA, and then treated with NS1619, DHEA, or both as described in the Supplemental Materials and Method, and figure legends.

### Statistical analysis

SigmaPlot version 13.0 (Systat Software, Inc., San Jose, CA, USA) was used to generate graphs. Statistical analysis was performed using the non-parametric Mann–Whitney rank-sum test. Results yielding *p*-values < 0.05 (*), < 0.01 (**), < 0.001 (***) were considered statistically significant. Survival curves were estimated with the Kaplan–Mayer method and groups were compared with the log-rank test. The groups were considered significantly different if the *p*-value was < 0.05.

### In vivo experiments

Five million PDX19 cells were transduced with a lentiviral vector coding for luciferase and inoculated into the tail vein of a NOD/SCID mouse. After 15 days, PDX19-luc cells were isolated from the spleen and injected into the tail vein of 20 NOD/SCID mice (1 × 10^6^ cells/mouse). One week later, the mice were divided into 2 groups of 10 and injected intraperitoneally with DEX (15 mg/kg) or with DEX (15 mg/kg) + NS1619 (0.1 mg/kg) every second day for the duration of the experiment. Blood samples were obtained weekly and analyzed for the presence of tumor cells, identified by flow cytometry after labeling with antibodies against human CD5 and CD7. For Kaplan–Meyer curves, an event was defined when CD5 + CD7 + cells represented at least 40% of total PBMC. Tumor cell growth was also monitored once a week by quantification of the luciferase signal using a Xenogen IVIS Spectrum In Vivo Imaging System. A pilot experiment carried out on groups of 6 mice showed that treatment every second day with NS1619 alone or DEX alone did not affect growth of PDX19 cells compared to 50% PEG-400 (vehicle).

## Electronic supplementary material

supplemental material
