# Supplementary material for: Selective killing of human T-ALL cells: an integrated approach targeting redox homeostasis and the OMA1/OPA1 axis
Source: Cell Death Dis. 2018 Aug 1;9(8):822. doi: 10.1038/s41419-018-0870-9 (PMC6070521; doi:10.1038/s41419-018-0870-9)
Supplement: Supplementary file 1 — supplemental material [file 41419_2018_870_MOESM1_ESM.pdf]

## SUPPLEMENTAL MATERIALS AND METHODS

*Mitochondrial ROS measurements*. Mitochondrial ROS were measured by flow cytometry in the gate of live cells using MitoSOXRed (Life Technologies) (44, 45). Cells were suspended at $1x10^6$/ml in complete RPMI plus 10% FCS and cultured at 37°C, 95% humidity and 5% $CO_2$ for 30 minutes in the presence of 2.6 μM MitoSOXRed. Cells were then analyzed at 15-minute intervals for a total time of 45 minutes with a BD LSRII flow cytometer using a 488-nm laser and a 582/15 nm bandpass filter. Drugs were added immediately before the first time point. The data were analyzed with Diva software (BD Biosciences). For each time point, the gate of live cells was used to calculate the difference between the mean fluorescence intensity (MFI) at each time point ($F_X$) and the MFI of the initial time point ($F_0$) divided by $F_0$ ($\Delta F/F_0$ ratios). In Figure S1C, TALL-1 cells treated with NS1619, DHEA, or NS1619+DHEA were stained with MitoSOXRed as described above immediately after drug addition (time 0) and after 24 and 48 hrs. Shown are resulting MFI.

*Immunofluorescence analysis.* Cells were seeded at 700,000/ml and treated with the indicated drugs. After 24 h, cells were fixed with 4% formaldehyde-PBS, permeabilized with PBS-0.01% NP40, and incubated for 1h at 37°C with rabbit anti-phosphoS40-Nrf2 antibody (1:100 in PBS, Abcam) and then incubated for 45 min at 37°C with Alexa 488-conjugated chicken anti-rabbit antibody (1:1000, Molecular Probes) and mouse anti-CD7 antibody conjugated with APC Vio770 (Miltenyi). Propidium iodide (PI, 0.1 μg/ml) and RNAseA (2 μg/ml) were added to stain the nucleus. Images were obtained with a Zeiss LSM510 confocal microscope equipped with argon (488 nm) and helium-neon (543 nm and 633 nm) laser sources and a 63X PlanFluo oil immersion objective; 1.2-μm optical slices were analyzed.

*Cell Death analysis*. Cell death was evaluated by staining cells with propidium iodide and analyzed by flow cytometry using a FacsCalibur and CellQuest software (BD Biosciences). Specific

Cell death was calculated using the formula [(% dead cells in the treated sample - % dead cells in the control sample) / (100 - % dead cells in control)] x 100.

*Immunoblot analysis*. Protein lysates were separated by SDS/PAGE, transferred to a nitrocellulose membrane and probed with the following antibodies: mouse anti-OPA1 (BD Biosciences), rabbit anti-Caspase 3 (Cell Signaling), rabbit anti-PARP1 (Cell Signaling), rabbit anti-β-actin (Sigma-Aldrich), mouse anti Hsp70 (BD Biosciences) and rabbit anti-vinculin (Cell Signaling), followed by incubation with an HRP-conjugated anti-mouse or anti-rabbit antibody (Pierce) and detection reagents (Super Signal West Femto Maximum sensitivity substrate, Thermo Fisher Scientific or LiteaBlot turbo substrate, Euroclone). Chemiluminescent signals were detected using a Cambridge UVITEC imaging system. The *cleaved OPA1 ratio* was calculated by dividing the signals of bands c, d, and e by the signals of all bands (see Fig. 4).

*Electron microscopy.* TALL-1 cells were seeded at 700,000 cells/ml and treated for 24 h with the indicated compounds. Cells were then washed in PBS and fixed with 2.5% glutaraldehyde in 0.1M sodium cacodylate buffer, pH 7.4 for 1 hour at 4°C, postfixed with a mixture of 1% osmium tetroxide and 1% potassium ferrocyanide in 0.1M sodium cacodylate buffer for 1 hour at 4°C and incubated overnight in 0.25% uranyl acetate at 4°C. After three water washes, samples were dehydrated in a graded ethanol series and embedded in an epoxy resin (Sigma-Aldrich). Ultrathin sections (60-70 nm) were obtained with an Ultrotome V (LKB) ultramicrotome, counterstained with uranyl acetate and lead citrate and viewed with a Tecnai $G^2$ (FEI) transmission electron microscope operating at 100 kV. Images were captured with a Veleta (Olympus Soft Imaging System) digital camera at 37000x magnification. At least 130 mitochondria for each sample were analyzed using ImageJ (IJ 1.46, NIH). Regions of Interest (ROIs) were manually traced and shape descriptors were obtained to analyze mean mitochondrial area and circularity ($4\pi$ x Area/Perimeter$^2$), which is a value between 0 (tubular) and 1

(circular). Computed data were analyzed using SigmaPlot to generate graphs and evaluate statistical significance (Mann-Whitney Rank Sum Test).

*Quantitative RT-PCR (qRT-PCR).* Total RNA was isolated using TRIZol (Life Technologies). RNA samples were treated with DNase I (Invitrogen) for 15 minutes at 37°C followed by addition of EDTA and incubation at 70°C for 10 minutes to inactivate the enzyme. The RNA was reverse-transcribed using SuperScript II reverse transcriptase (Invitrogen) and random hexamers. Aliquots of the resulting cDNA were PCR-amplified by using SYBR Green (Roche) and the following primers: β2-microglobulin-s: (TGACTTTGTCACAGCCCAAG); β2-microglobulin-as: (TTCAAACCTCCATGATGCTG); TRAILR2-s: (CCAGGTGTGATTCAGGTGAA); TRAILR2-as: (CCCCACTGTGCTTTGTACCT); OMA1-s (CATTGTAGGCAGGGGCATAA); OMA1-as (CACCACAAAGAGCAATCCAAAA). The OPA1 cDNA was amplified using primers OPA1-s (TCAAGAAAAACTTGATGCTTTCA), OPA1-as (GCAGAGCTGATTATGAGTACGATT) and probe #2 from the Roche Universal Probe Library. NRF2 target genes were analyzed using TaqMan Gene Expression Assays (ThermoFisher Scientific). The PCR reactions were performed in a LightCycler 480 thermal cycler (Roche) according the manufacturer's protocol. β2-microglobulin was used as a housekeeper mRNA for the calculation of relative expression values.

*RNA silencing experiments.* Five million TALL-1 cells were mixed with 312.5 pMol of siRNA (Oma1#S41777 or Silencer Negative Control 1 #4390843, Thermo Scientific) and electroporated with a Neon transfection system (Thermo Scientific) using a single 1410V- 30 msec pulse. Following overnight culture in complete RPMI with 30% FCS, the electroporated cells were seeded at $7X10^5$/ml in complete RPMI-10% FCS and treated as indicated in the figure legends.

## SUPPLEMENTAL TABLES

| SAMPLE | TTL | Risk | Phenotype | PPR/PGP | Notch | FBW7 |
|---|---|---|---|---|---|---|
| PDX6 | 132 | MR | T III | PGR | WT | WT |
| PDX8 | 72 | MR | T III | PPR | HD+PEST mut | WT |
| PDX9 | 70 | HR | T-I-II | Relapse | WT | WT |
| PDX11 | 83 | MR | T-I-II | PPR | HD mut | WT |
| PDX15 | 48 | HR | T-IV | PPR | WT | WT |
| PDX19 | 119 | MR | T-I-II | PGR | HD+PEST mut | WT |
| PDX29 | 159 | HR | T -III | PPR | WT | WT |
| | | | | | | |

**Table S1. Clinical and molecular characteristics of T-ALL PDX.**

TTL: time to leukemia; high risk (HR) or medium risk (MR) to relapse; PPR: prednisone poor responder; PGR: prednisone good responder.

| Patient | Sex | Age | Phenotype | Prednisone response |
|---|---|---|---|---|
| 1 | M | 13 | ETP | PPR |
| 2 | F | 9 | T-I-II | PPR |
| 3 | M | 12 | T-III | N/A |
| 4 | M | 9* | T-III | Relapsed |
| 5 | M | 13 | T-III | PPR |
| 6 | M | 11 | T-I-II | PGR |
| 7 | M | 5 | T-III | PGR |
| 8 | M | 16 | T-III | PGR |
| 9 | M | 14 | T-I-II | PPR/Deceased |
| 10 | M | 12* | T-III | Relapsed |
| 11 | M | 5 | T-I-II | PGR |
| 12 | M | 12 | T-III | PGR |
| 13 | M | 15 | T-III | PPR |
| 14 | M | 11 | T-I-II | PPR |
| 15 | F | 11* | T-I-II | Relapsed |
| 16 | M | 14 | T-III | PGR |

**Table S2. Clinical features of T-ALL patients classified by sex, age, tumor phenotype and response to prednisone.**

Age indicates years at diagnosis, except where marked by*, which indicates age at relapse.

## SUPPLEMENTARY FIGURES

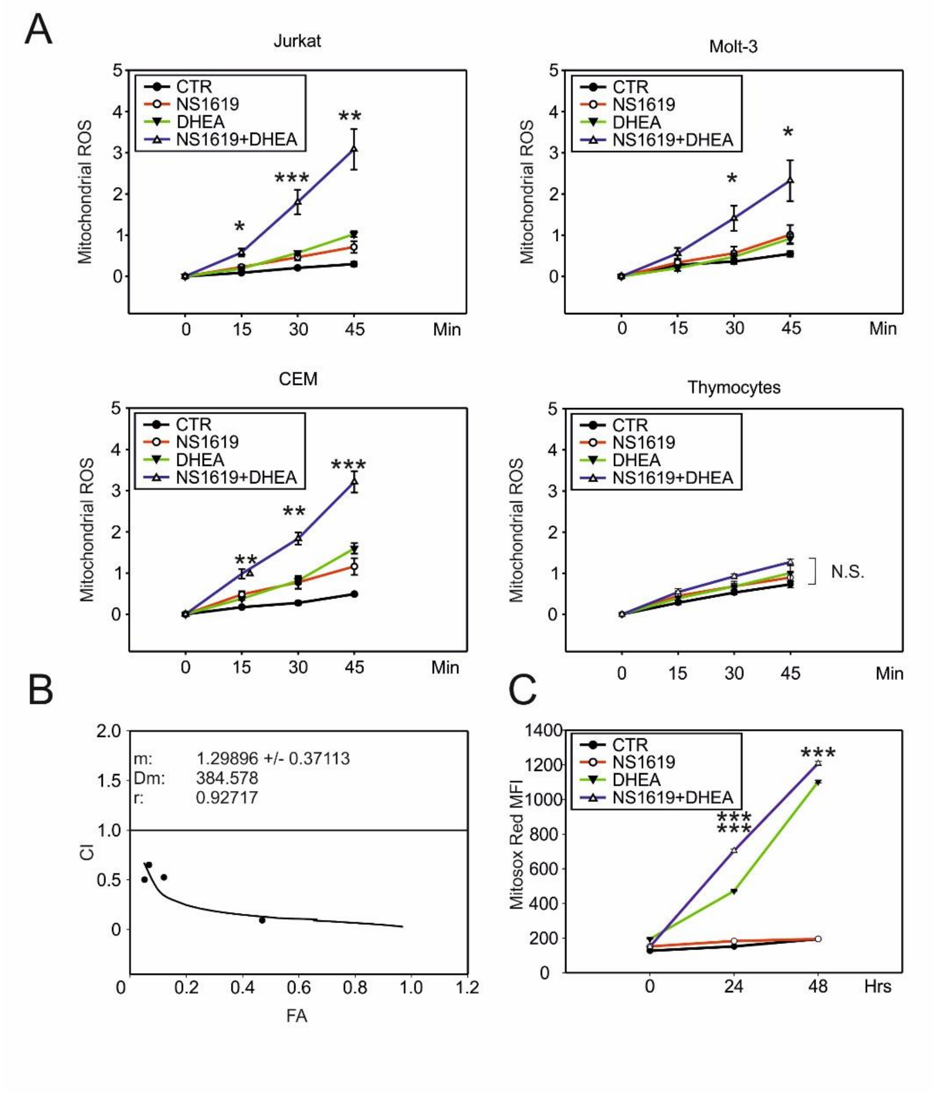


**Figure S1. Effects of NS1619 and DHEA on mitochondrial ROS (mtROS).**

(**A**) mtROS accumulation was measured using MitoSOXRed and flow cytometry and expressed as $\Delta F/F_0$ in T-ALL cell lines Jurkat, Molt-3, CEM, and primary normal thymocytes. Responses to NS1619 (red), DHEA (green) or NS1619+DHEA (blue) are shown for a total time of 45 min. Mean values and standard error bars from 3 independent experiments (3 replicates each) are shown. (**B**) The combination index of NS1619 and DHEA was calculated using the CompuSyn software. The FA (Fraction affected) represents the ΔF/F0 of MitoSOXRed at 60 min. (**C**) MFI of MitoSox Red accumulation in TALL-1

cells treated with NS1619 (red), DHEA (green) or NS1619+DHEA (blue) for 0, 24 and 48 hours. Mean values and standard error bars from 3 replicates are shown.

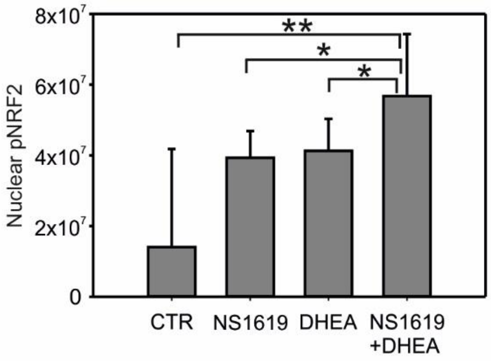


**Figure S2. Quantification of nuclear pNRF2.**

Dot plots from 15 randomly selected immunofluorescence fields for each treatment were used to calculate phospho-NRF2-PI colocalization by multiplying the MFI of the phospho-NRF2 signal by the number of pixels in Region 3 (see Fig. 2a). The graph shows mean values and error bars of nuclear NRF2 in TALL-1 cells following the indicated treatments.

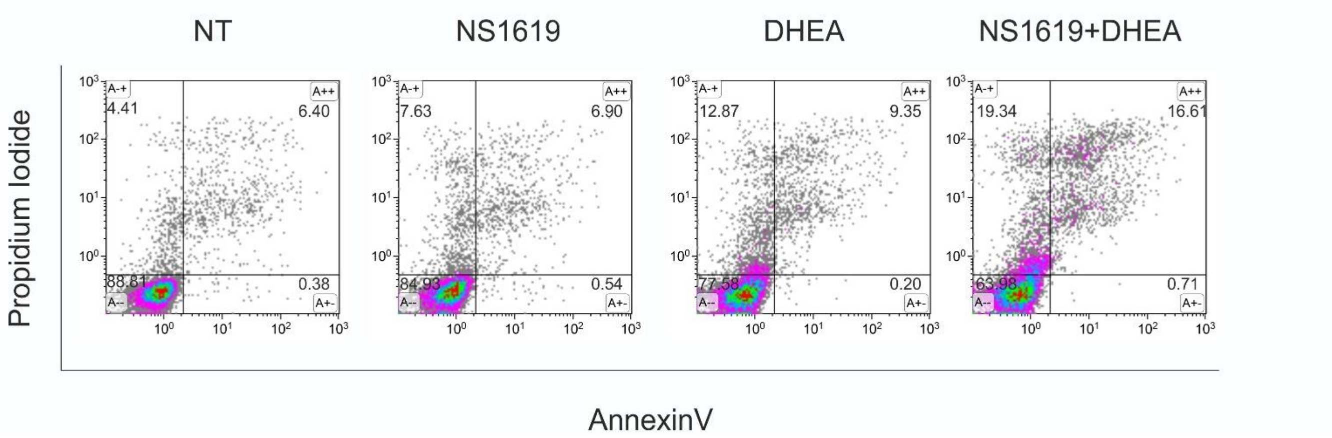


**Figure S3. Treatment with NS1619 plus DHEA induces apoptosis in TALL-1 cells.**

Annexin V/PI staining of TALL-1 cells after 24 h of treatment with NS1619 and DHEA showed that the two drugs increased the percentage of cells positive for Annexin V and PI (A++ quadrant). Samples were analyzed using a FACSCalibur flow cytometer (BD Biosciences) and Kaluza Analysis Software (Beckman Coulter Life Sciences).

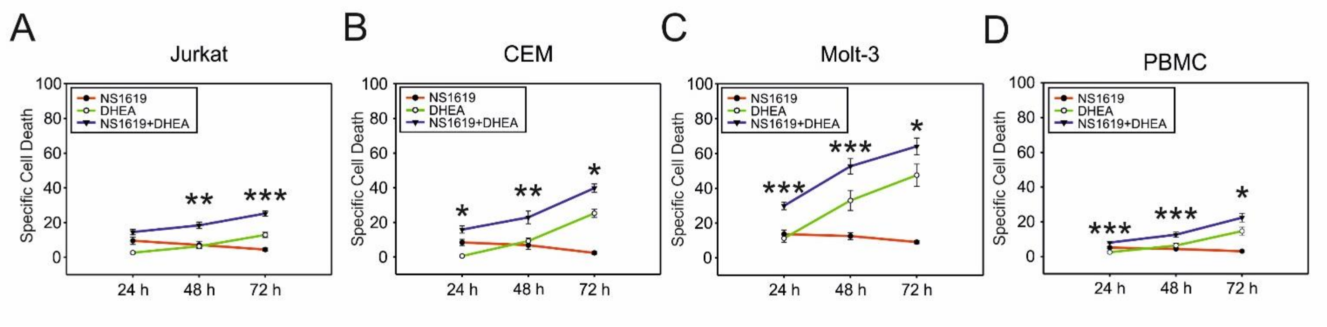


**Figure S4. NS1619 and DHEA induce death in T-ALL cell lines.**

Specific cell death of Jurkat (**A**), CEM (**B**), Molt-3 (**C**), and normal PBMC (**D**) after 24, 48 and 72 h of treatment with NS1619 (red), DHEA (green) or NS1619+DHEA (blue); mean values and standard error bars from 3 independent experiments, 3 replicates each are shown.

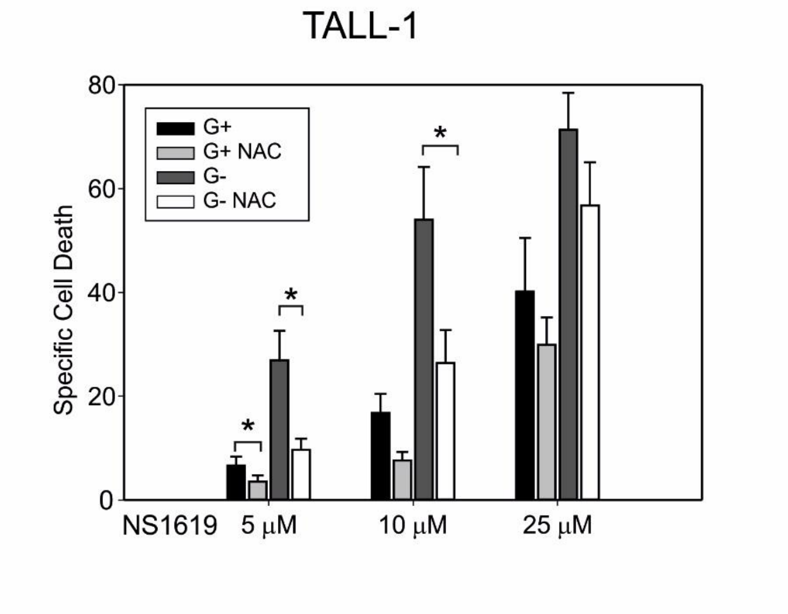


**Figure S5. NS1619-induced cell death is enhanced by glucose starvation.**

Effects of NS1619 on specific cell death in the presence or absence of glucose were measured. Where indicated, the cells were pre-incubated with 500 μM N-acetyl-L-cysteine (NAC, Sigma-Aldrich) for 16 h (light gray and white bars). Mean values of specific cell death and standard error bars from 4 independent experiments (2 replicates each) are shown.

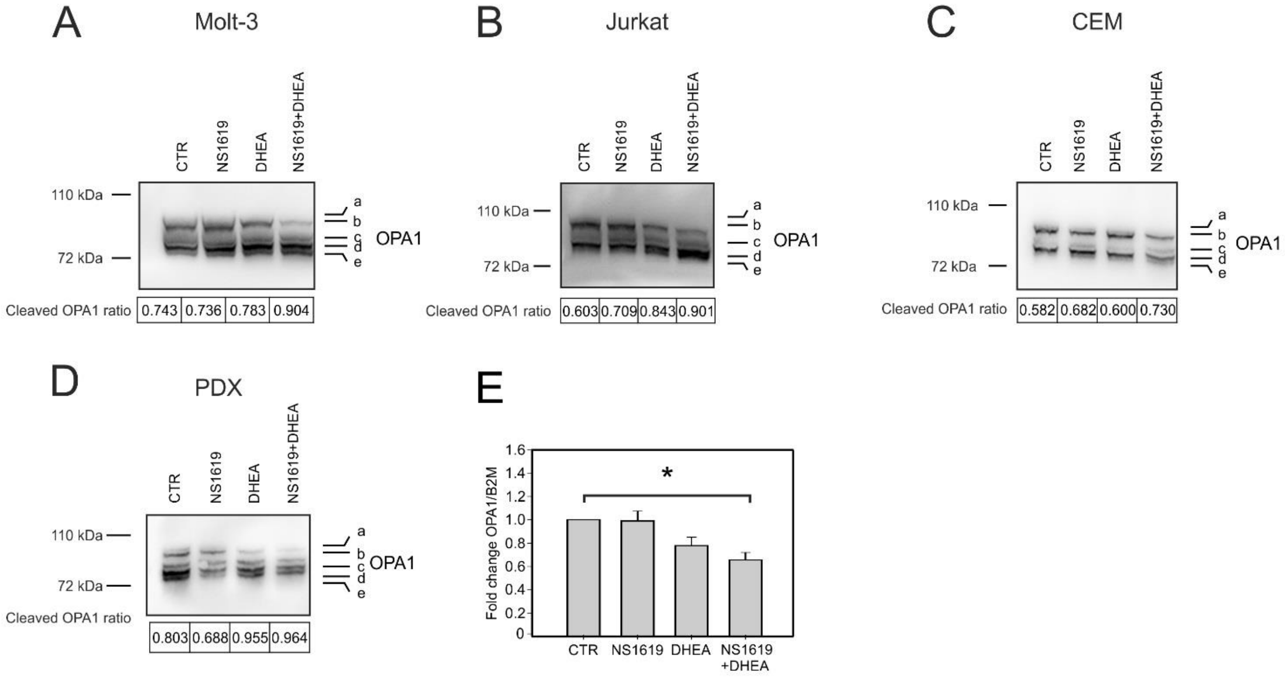


**Figure S6. Effects of NS1619 and DHEA on OPA1 cleavage and expression.**

**A-D**. Immunoblot analysis of a representative experiment showing cleavage of OPA1 following 24 h of treatment with NS1619 and/or DHEA in Molt-3 (**A**), Jurkat (**B**) and CEM (**C**) cell lines and PDX (**D**). Cleaved OPA1 ratios are indicated below each gel lane. The positions of protein markers (SHARPMASS VI Prestained Protein Marker, Euroclone) are shown to the left of the images.

**E**. Quantitative RT-PCR analysis of OPA1 mRNA expression after 24 h of treatment with NS1619, DHEA or NS1619+DHEA in TALL-1 cells. Data were normalized using the B2M mRNA as a control and scaled against the non-treated control. Mean values and standard error bars from 5 experiments are shown.

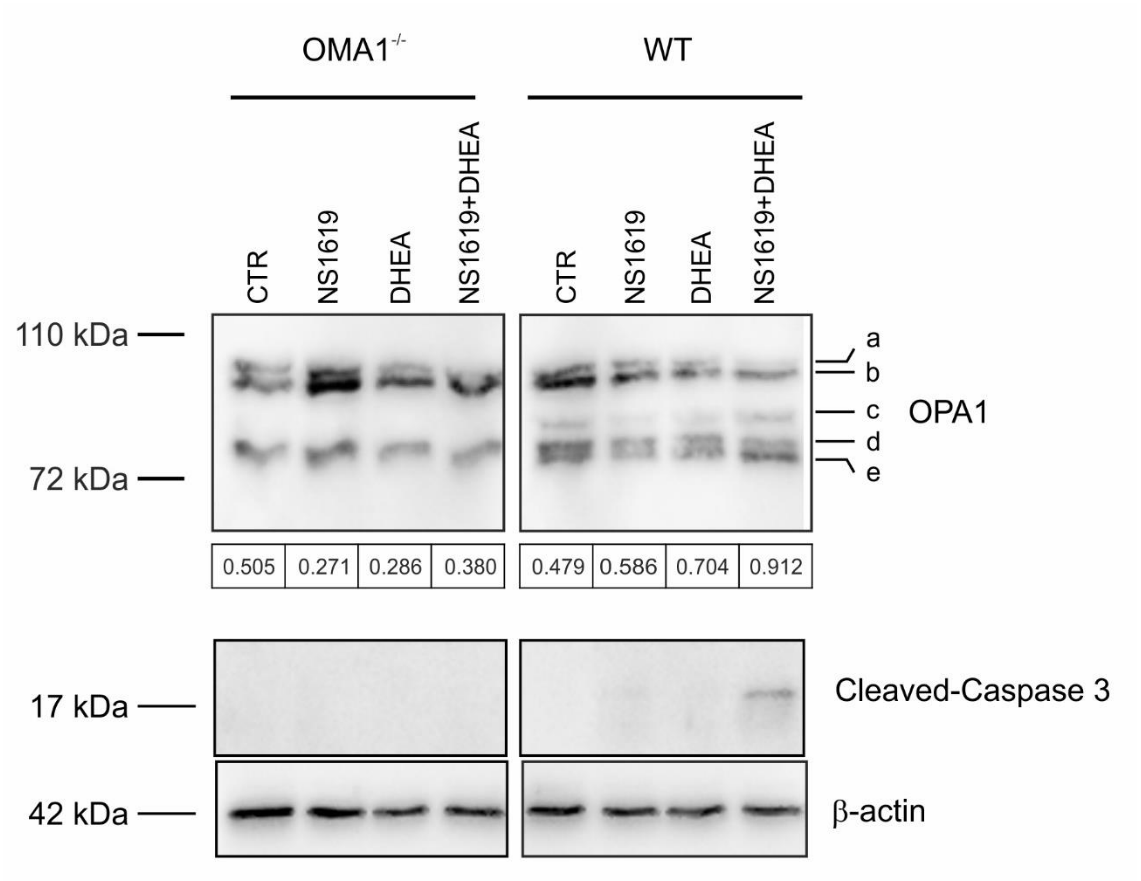


**Figure S7. Effects of NS1619 and DHEA on OPA1 cleavage in OMA1-/- cells.**

Immunoblot analysis of a representative experiment showing cleavage of OPA1 and Caspase 3 in OMA1 knockout (OMA1$^{-/-}$) and OMA1 wild type (WT) fibroblasts following 24 h of treatment with NS1619 and DHEA alone or in combination. β-actin was used as a loading control.

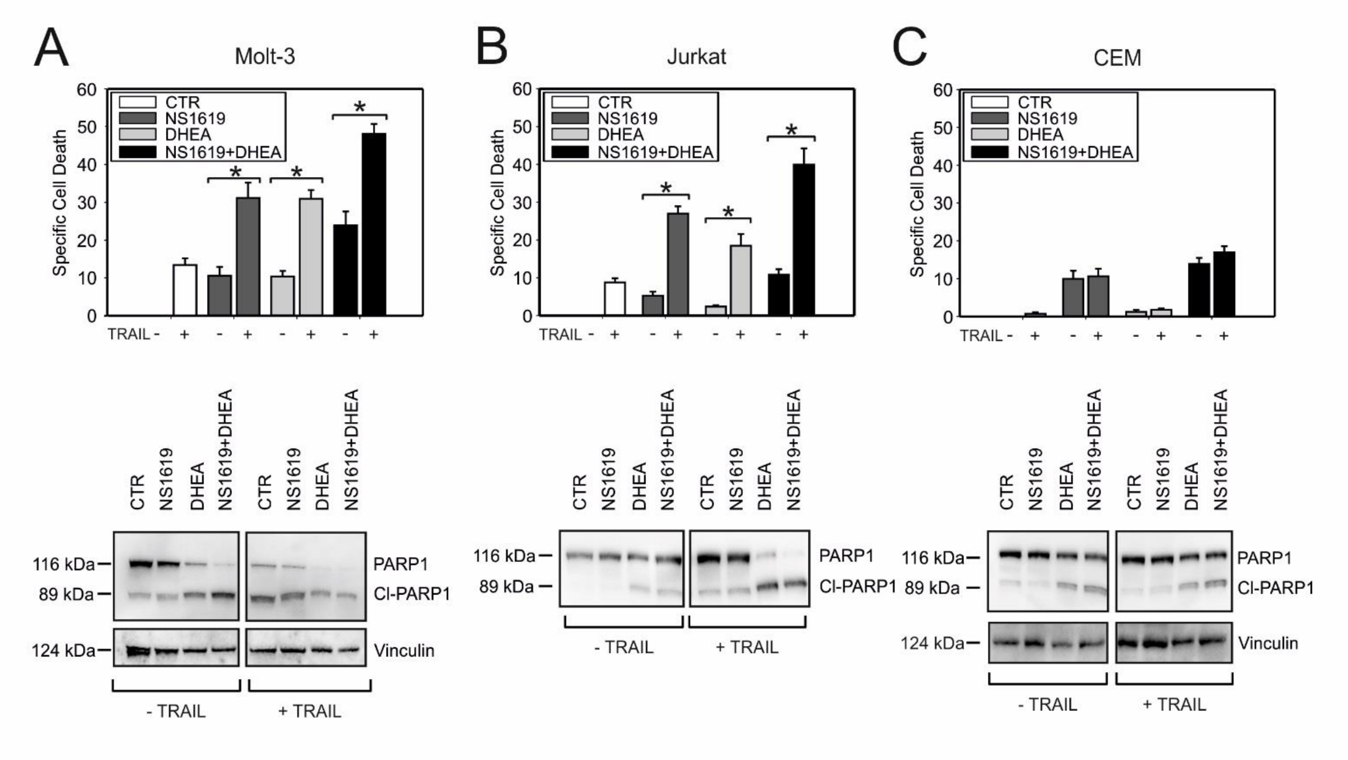


**Figure S8. NS1619 and DHEA sensitize T-ALL cell lines to TRAIL-induced death.**

**A-C (upper panels)**. Specific cell death upon 24 h of treatment with NS1619, DHEA or NS1619+DHEA in the absence (-) or presence (+) of TRAIL in Molt-3 (**A**), Jurkat (**B**), and CEM (**C)** cells. Mean values and standard error bars from 3 independent experiments are shown.

**A-C (lower panels).** Immunoblots (composite figure) of representative experiments showing changes in PARP1 cleavage in the 3 T-ALL cell lines after 24 h of treatment with combinations of NS1619, DHEA and TRAIL. Vinculin was used as a loading control for all cell lines except for Jurkat, which do not express it.

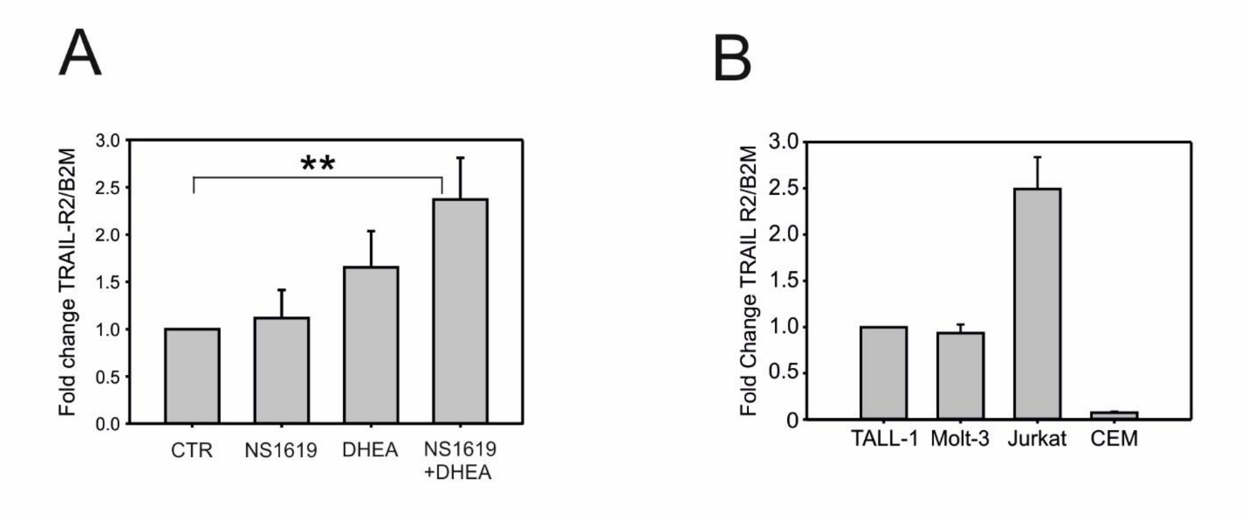


**Figure S9. Effects of NS1619 and DHEA on TRAIL-R2 expression**

**A**. Quantitative RT-PCR analysis of TRAIL-R2 mRNA expression in TALL-1 cells after 24 h of treatment with NS1619 and/or DHEA compared to non-treated cells (set at 1). Data were normalized using B2M mRNA as a housekeeping control. Mean values and standard error bars from 6 independent experiments are shown.

**B**. Quantitative RT-PCR analysis of TRAIL-R2 mRNA expression in the indicated T-ALL cell lines. Data were normalized using B2M mRNA as a housekeeping control and scaled against TALL-1 (set at 1). Mean values and standard error bars from 6 replicates are shown.

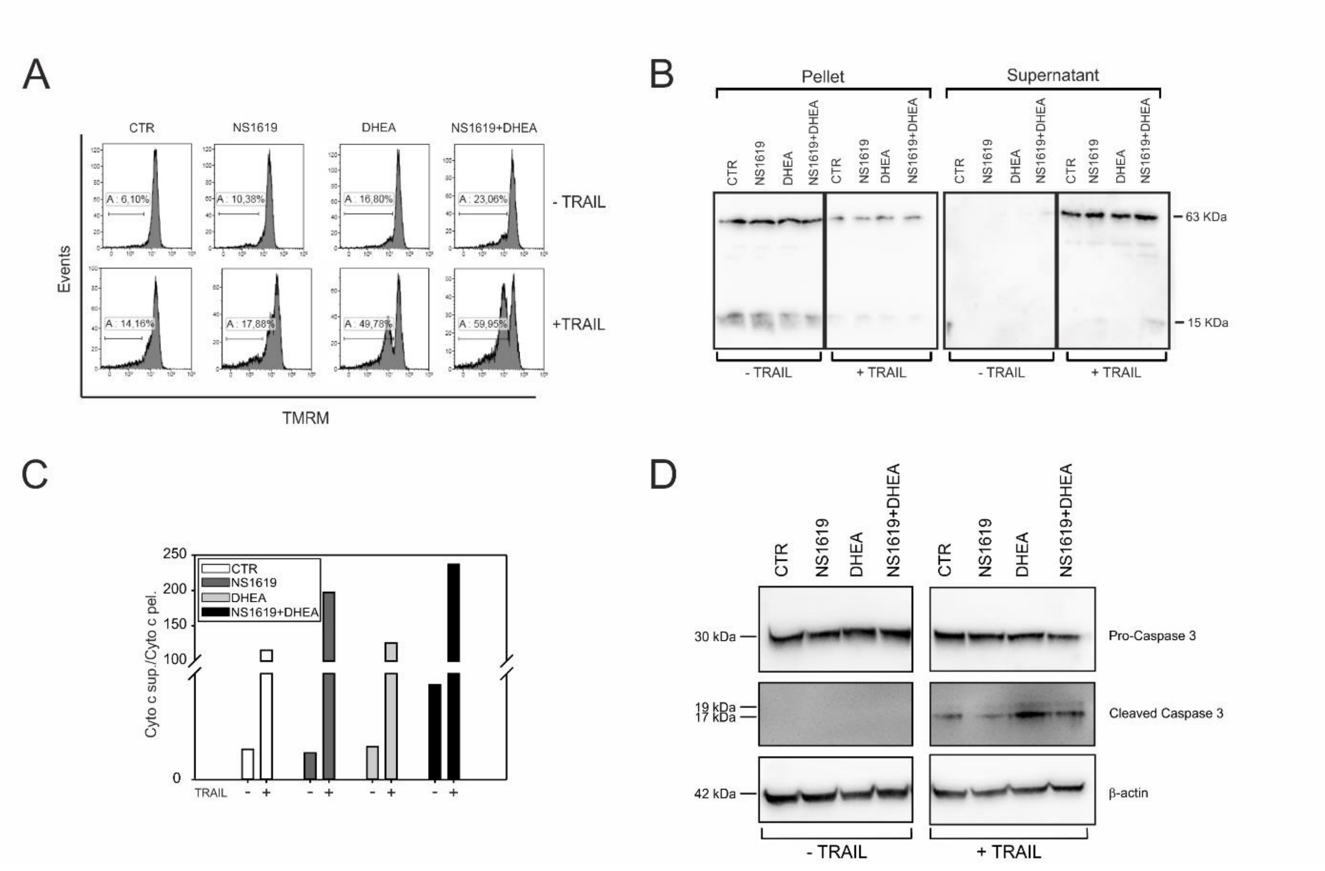


**Figure S10. Effects of NS1619, DHEA and TRAIL on mitochondrial targets**

**A**. Mitochondrial membrane potential was measured with TMRM and flow cytometry. Shown are percentages of TALL-1 cells with depolarized mitochondria (gate A) after 24 h of treatment with NS1619, DHEA or NS1619+DHEA in the absence or presence of TRAIL.

**B**. Immunoblot analysis (composite figure) of cytochrome c in Supernatant (cytosolic) and Pellet fractions of TALL-1 cells following treatment for 16 h with the indicated drug combinations. Cytochrome c was detected as two major bands compatible with monomeric (15 kDa) and tetrameric (63 kDa) forms of the protein.

**C**. Quantification of cytochrome c in the Pellet and Supernatant fractions. Cytochrome c release was calculated as the ratio between the signal of cytochrome c (monomer and tetramer) in the Supernatant and Pellet in TALL-1 cells treated with the indicated drug combinations.

**D**. Immunoblot analysis (composite figure) of a representative experiment showing cleavage of Caspase 3 in TALL-1 cells upon treatment for 16 h with combinations of NS1619, DHEA and TRAIL. β-actin was used as a loading control.

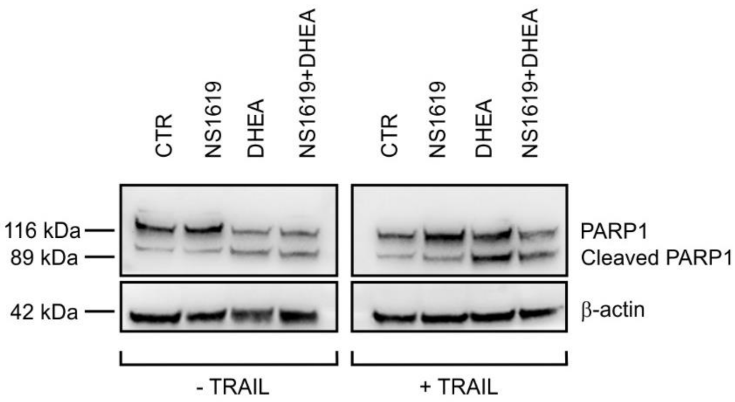


**Figure S11. Effects of NS1619, DHEA and TRAIL on PARP1 cleavage in PDX cells**

Immunoblot analysis of a representative experiment showing PARP1 cleavage in PDX9 cells following 16 h of the indicated treatments. β-actin was used as a loading control.

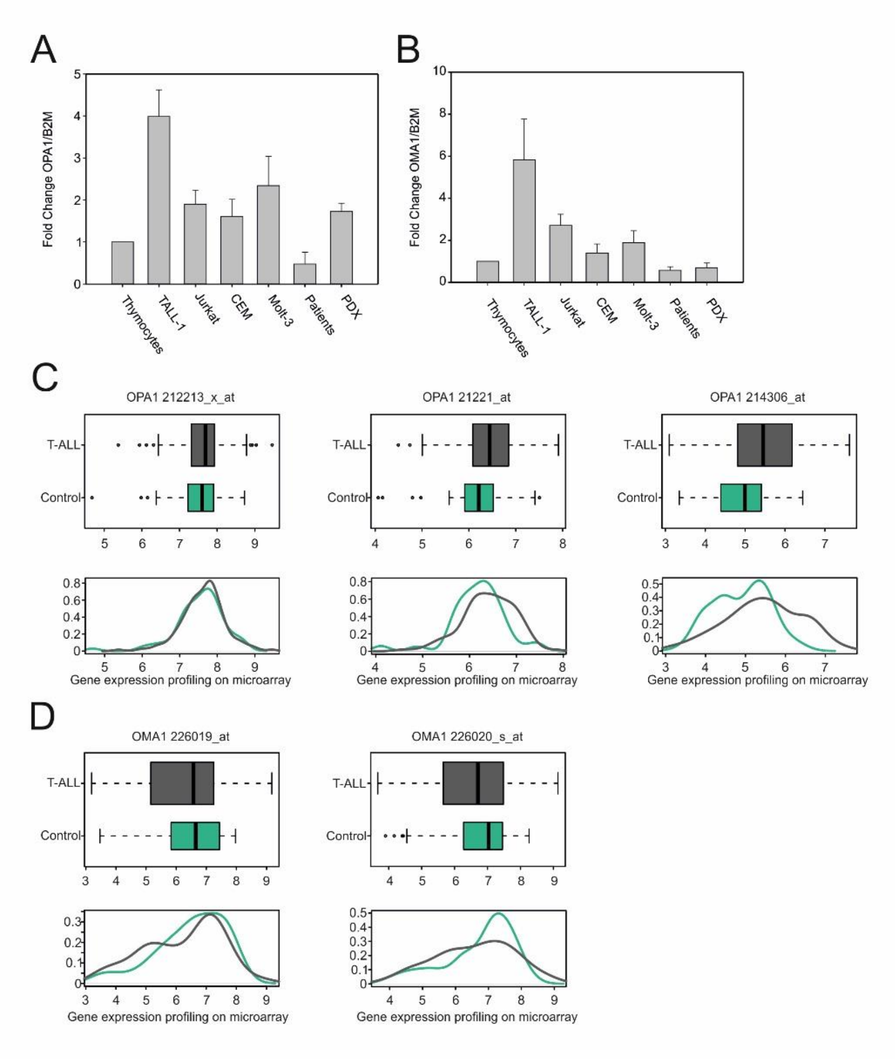


**Figure 12. OPA1 and OMA1 expression in T-ALL cells.**

Quantitative RT-PCR analysis of OPA1 **(A)** and OMA1 **(B)** mRNA expression in the indicated T-ALL cells and thymocytes. Data were normalized using B2M mRNA as a housekeeping control and scaled against values obtained for thymocytes. Mean values and standard error bars from 3 independent assays are shown. Gene expression profiling of OPA1 (**C**, three probe sets) and OMA1 (**D**, two probe sets) are represented as box plots and distribution curves. Data were obtained using the Leukemia Gene Atlas (LGA) public platform. Mean values and standard error bars from 174 primary T-ALL samples and 73 non-leukemic bone marrow samples are shown.
